# ^68^Ga-DOTATATE Radioisotope scan to detect neuroendocrine tumors; A Cross-Sectional Study

**DOI:** 10.22038/AOJNMB.2021.56971.1397

**Published:** 2022

**Authors:** Abtin Doroudinia, Habib Emami, Mahsa Sadat Hosseini

**Affiliations:** Chronic Respiratory Diseases Research Center, National research institute of tuberculosis and lung diseases, Shahid Beheshti University of Medical Sciences, Tehran, Iran

**Keywords:** ^68^Ga-DOTA-peptide PET/CT, Neuroendocrine tumors Diagnosis

## Abstract

**Objevtive(s):**

Neuroendocrine tumors are a heterogeneous group of neoplasms that arise from the peptide-producing cells of the neuroendocrine system. Different functional imaging methods have been suggested to diagnose NETs. There is still not enough evidence to recommend ^68^Ga-DOTATATE as a standard diagnostic tool in NETs. Therefore, the aim of this study was to assess the value of ^68^Ga-DOTATATE scan in detecting NETs.

**Methods:**

This was a cross-sectional study. All patients with a pathologically confirmed NET tumor referred to Masih Daneshvari Hospital affiliated to Shahid Beheshti University of Medical Sciences entered the study. Patients underwent a ^68^Ga-DOTATATE PET/CT. All statistical analysis were performed by SPSS software version 18.

**Results:**

Forty patients with a mean age of 48.1±15.80 years entered the study. Twenty-one (52.5%) were male and 19 (47.5%) female. In the studied patients, neuroendocrine tumor was present in 19 cases (47.5%) in pancreas and gastrointestinal tract, 9 (22.5%) in lung, 3 (7.5%) in mediastinum and adrenal gland, 6 cases (5%) in liver and 3 other sites. There was no significant association between mean age and gender with primary location of the tumor. The mean SUV_max _was 11.62±20.02 and the the mean tumor size was 38.25±31.35 mm. The mean size of the metastasis was 40.55±24.53 mm. The mean percentage of ki-67 was 12.54±18.40. There was no significant correlation between SUVmax of the lesion and age (r=0.063, P=0.701), tumor size (r=-0.63, P=0.067) or Ki-67 (r=0.011, P=0.960). In 20 cases, metastases were reported, of which 14 were (70%) in the liver, 3 in the lungs (15%), 2 in the gastrointestinal and cervical lymph nodes, and 1 in the bones and pancreas(%5).

**Conclusion:**

^68^Ga-DOTA-peptide PET/CT could find the primary or metastasis sites of NETs with good quality images. In general, this modality can enhance the management in patients with NETs.

## Introduction

 Neuroendocrine tumors are a heterogeneous group of neoplasms that arise from the peptide-producing cells of the neuroendocrine system (1, 2). Histologically, a distinctive feature of this type of tumor is the presence of endocrine tissue markers such as chromographin A, synapto-physin, and neuron-specific enolase that can be used for diagnostic purposes (3). 

 Most of these tumors occur in the gastrointestinal tract (66%) and then the lungs (25%) and are very rare in other organs such as the adrenal medulla, pituitary, parathyroid and thyroid (4, 5). Neuroendocrine neoplasms have variable symptoms, such as biological signs of excessive secretion of bioactive amines. But most of these tumors are dysfunctional and are usually discovered when they are enlarged or

have metastasized to the liver (6, 7). 

 Most NETs express somatostatin receptors (SSTRs), which can be used as targets for radionuclide imaging and therapy. Planar images and SPECT or SPECT/CT, Scintigraphy SSTR show limitations that can reduce the diagnostic effect. This is mostly due to the high physiological absorption including in the liver as well as lack of detection of smaller lesions due to the sub-optimal physical properties of radiopharma-ceuticals and the low resolution of gamma cameras (8, 9). Radiotherapy is an emergency treatment for neuroendocrine tumors. It is a radioconjugate containing tyr3-octreoate or TATE, which has a very high affinity for the SSTR2 type, and is present in cell membranes of various types of NETs. This allows the visualization of SSTR positive cells during imaging. SSTR subtypes have been shown to be abundant in NETs and their metastases, while most other natural tissues express low levels of SSTR subtypes (10, 11).

^68^Ga-DOTATATE is a labeled SSA for use in PET or PET/CT for the localization of SSR-positive NETs in adults and children. According to previous studies, ^68^Ga-DOTATATE PET/CT showed a high sensitivity (above 94%) and specificity (above 92%) for localization of NET lesion, which was most accurate for tumors of midgut origin (12).

 There is still not enough evidence to recommend ^68^Ga-DOTATATE as a standard diagnostic tool in NETs. Therefore, the aim of this study was to assess the value of ^68^Ga-DOTATATE scan in detecting NETs.

## Methods

 This was a cross-sectional study. All patients with confirmed histopathologic NET referred to Masih Daneshvari Hospital affiliated to Shahid Beheshti University of Medical Sciences entered the study. ^68^Ga/^68^Ge generator was used as the Ga-68 source which was attached to DOTATATE module. Primary peptide precursor was DOTATAE-HBERD which was labeled with Ga-68 using required solvents in suitable conditions. Finally, a sample of the product was checked in the quality control laboratory. General appearance, activity and pH, radionuclide purity, radiochemical features were checked using GAMA spectroscopy and chromatography techniques (TLC, HPLC, GC). Also, endotoxin test and microbial control was performed. Production processes and quality control were performed in the Iranian Atomic Energy Organization. 

 Then appropriate parameters related to recon-structing images using ^68^Ga- DOTATATE, 

suitable for imaging protocol was determined. PET/CT was performed using GE healthcare ADW 4.5 workstation. Exclusion criteria were patients who had received octreotide, chemo-therapy or radiotherapy within the last one month. Imaging was performed using Discovery 690 GE PET/CT, equipped with 64 slice CT scanner and Time of Flight (TOF) technology.

 Pearson correlation coefficient was used to evaluate the correlation of dependent variables. Chi-square test was used to compare qualitative variables and independent T test for quantitative ones. All analyzes were performed by SPSS software version 18 (SPSS Inc. Chicago, Il, The USA). 

 All steps were explained to all participants before enrollment and a written informed consent was obtained from all individuals. Also, all costs were afforded by the research team. All the study steps were performed according to the declaration of Helsinki. Patients were free to leave the study at any time without affecting their standard treatment protocol.

## Results

 Forty patients entered the study. The mean±SD age of patients was 48.1±15.80 years. Twenty-one (52.5%) patients were male and 19 (47.5%) female. The mean±SD age of men was 53.52±12.97 years. The mean±SD age of women was 42.10±16.79 years (Table 1). The mean time elapsed from the last chemotherapy and radiotherapy treatment session was 3.21±0.45 months.

**Figure 1:**
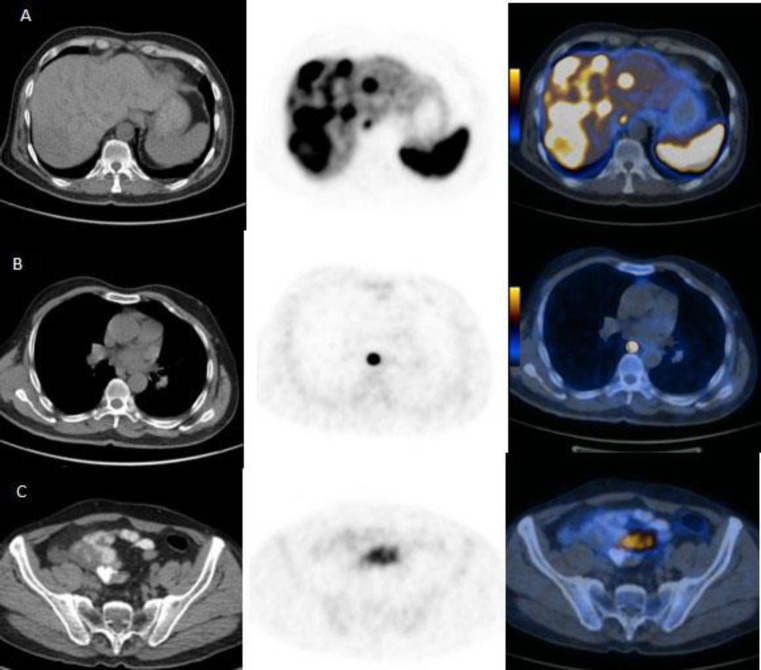
Rows A, B and C shows CT scan, PET and PET-CT image of Liver, Mediastinum and Pelvis involvement by tumor, respectively

 Neuroendocrine tumor was present in 19 cases (47.5%) in pancreas and gastrointestinal tract, 9 (22.5%) in lung, 3 (7.5%) in mediastinum and adrenal gland, 6 cases (5%) in liver and 3 other sites (Figure 1). There was no meaningful statistical difference between age and gender with the tumor site (P values; 0.154 and 0.192, respectively). 

 The obtained SUV_max_ for the initial location of the tumor was 7.67±2.41. The mean tumor size was 38.25±31.35 mm. There was no significant correlation between SUV and age (r=0.063, P=0.701). There was no significant correlation between SUV and the tumor size (r=-0.63, P=0.067). There was no correlation between SUV size and Ki-67 (r=0.011, P=0.960).

 The mean percentage of ki-67 in patients was 12.54±18.40. The rate of differentiation was well in 29 patients (72.5%) and poor in 7 patients (17.5%). Ki-67 information was not available in all patients. In 20 cases, metastases were reported, of which 14 were (70%) in the liver, 3 in the lungs (15%), 2 in the gastrointestinal and cervical lymph nodes, and 

1 in the bones and pancreas (5%). The SUV_max _obtained from the metastases in the scan was 40.40±48.75. 

 The mean size of the metastasis was 40.55±24.53 mm.

**Table 1:** A summary of demographic characteristics of the study participants

**Variable **	**Value**
**Age, (Mean±SD)**	48.1±15.80
**Gender **	
Male, %	21 (52.5%)
Female, %	19 (47.5%)
**Tumor location**	
Pancreas and GI	19 (47.5%)
Lungs	9 (22.5%)
Mediastinal and adrenal glands	3 (7.5%)
liver	6 (5%)
**Tumor size, (Mean±SD)**	38.25±31.35
**ki-67**	12.54±18.40
**Tumor differentiation**	
Well-differentiated	29 (72.5%)
Poorly-differentiated	7 (17.5%)

## Discussion

 Neuroendocrine tumors (NETs) are a large group of neoplasms usually originated from neuroendocrine cells of the GI (gastrointestinal) tract. Somatostatin receptors are expressed in the surface of well-differentiated NETs tumors, which has been targeted for diagnostic approaches using radiolabeled somatostatin analogues (13, 14). Different diagnostic tools have been emerged for the diagnosis of primary site of NETs or their metastasis. Such a diversity is due to the variety of NETs and the location and also different used somatostatin analogues (15, 16). Therefore, there is still no consensus regarding the gold standard diagnostic modality in NET of different origins. Somatostatin is a small cyclic neuropeptide found in neurons and neuroendocrine cells and has a high density in the brain, peripheral neurons, pancreas, endocrine glands and gastrointestinal tract. Because the stability of naturally secreted somatostatin is very low, synthetic analogues have been developed with much greater stability (17, 18).

 Neuroendocrine tumors account for 0.66% of all malignancies in the United States and their incidence is increasing at a rate of 3 to 10% per year (19). This increase is probably related to the introduction of more sensitive diagnostic tools and increased awareness of physicians and pathologists (20). To date, surgical excision of NETs is the standard of care to remove the primary site or metastases.

 In this study, we investigated the role of ^68^Ga-DOTATATE scan in the diagnosis of endocrine tumors. The most common site of primary endocrine tumor was in the pancreas and gastrointestinal tract. Metastasis was observed in 20 patients, and the most affected organ was liver. In the study of Angelena Crown et al. (21) in 2019, the most common sources of tumors were the GI tract including pancreas (75%), unknown primary (13%), lung (8%) and thymus (2%). In our study, the highest NET origin was from the gastrointestinal tract and pancreas which is in line with our findings.

 Data published from the German NET registry on a large number of patients (860 patients) also showed that the most common sites of primary NETs were pancreas and midgut. 10-year survival of patients in this study was 70%. They found that those patients who underwent a surgical resection survived more than others with a statistically significant difference. They finally concluded that surgical resection should be considered for all patients with a resectable tumor (22). This national data registry could be a sample for other countries to find regional tumor habits. This would help for earlier diagnosis and better management of NETs tumor. Unfortunately, we do not have such a data registry in Iran which is suggested to be established by multidisciplinary management. 

 In our study, ^68^Ga-DOTATATE scans correctly identified the location of the primary tumor and metastasis in 5 of 6 patients who were referred for metastatic (sensitivity of 83.33%, specificity 69.3%) and in 24 of 34 patients who were referred for endocrine tumor primary site evaluation (sensitivity of 70.58%, specificity 61%). Our study is in agreement with the literature that well-differentiated NETs have higher SUV_max_ compared with poorly differen-tiated ones.

 Michael S Hofman et al. declared that the ^68^Ga-DOTATATE method provides more diagnostic information in a large number of patients. They suggested that this method can replace In-111 octreotide, because it has a higher accuracy, is done faster and has a low radiological radiation rate (23). In a study by Zeynep Gözde kanzkan and colleagues in 2013 ^68^Ga-DOTATATE was compared to In‐111 octreotide to determine the location of an ectopic ACTH-producing tumor. This study was performed on 19 patients during 12 years. They suggested that PET/CT imaging with ^68^Ga-DOTATATE is a new method for detection of ACTH-producing tumors with high sensitivity and specificity (24). Our study lacked a comparative design to assess two imaging modalities, however, such a study using ^68^Ga-DOTATATE to detect NETs has not been performed to our knowledge in our region. Therefore, our study can persuade other centers to use ^68^Ga-DOTATATE to enhance the care provided to NETs patients.

 Furthermore, Frilling et al. (25) also reported that therapeutic decisions could be changed using ^68^Ga-DOTATOC PET/CT in patients who have previously undergone other imaging modalities. They claimed that in almost 60% of the cases therapeutic management was changed according to new findings of somatostatin analogues. This highlights the importance of somatostatin analogues scan compared to CT or MRI. In fact, ^68^Ga-DOTATOC PET/CT found the primary site of tumor not detected by other conventional imaging modalities.

 On the other hand, a meta-analysis by Jigang Yang et al. in 2014 compared ^68^Ga-DOTATOC and ^68^Ga-DOTATATE methods in PET imaging to detect NET tumors. In this study, 416 patients were studied. The sensitivity of ^68^Ga-DOTATOC and ^68^Ga-DOTATATE were 93% and 96%, respectively. The specificity of ^68^Ga-DOTATOC and ^68^Ga-DOTATATE were 85% and 100%, respectively (26). They claimed the high value of somatostatin scan in detecting the tumor site, which is in line with our study.

 A study by Delpassand et al. from Texas assessed the appropriate dosage of radiolabeled analogue to find the least required dosage of ^64^Cu-DOTATATE to yield high-quality PET/CT images. They reported that a dose of 148 MBq (4.0 mCi) ^64^Cu-DOTATATE is adequate to produce high quality PET/CT images (27).

 We had some limitations. Our sample size was quite small which is due to rarity of this type of tumor. However, comparative studies with different imaging modalities could yield the superiority of ^68^Ga-DOTATATE in detection of NETs in further investigations. Therefore, further larger multi-centric clinical trials are required to evaluate the value of ^68^Ga-DOTA-peptide PET/CT in NETs of different organs. A national data registry is also suggested to be stablished. This helps for better management of patients and early detection of metastasis to improve their survival and quality of life. 

## Conclusion

^68^Ga-DOTA-peptide PET/CT could find the primary or metastasis sites of NETs with good quality images. In general, this modality can improve the management of patients with NETs.

## Funding and Support

 This study was financially supported by Shahid

Beheshti University of Medical Sciences.

## References

[1] Kulke MH, Benson AB, Bergsland E, Berlin JD, Blaszkowsky LS, Choti MA (2012). Neuro-endocrine tumors. Journal of the National Comprehensive Cancer Network..

[2] Clark OH, Ajani JA, Benson AB, Berlin JD, Blaszkowsky LS, Byrd D (2009). Neuro-endocrine tumors. JNCCN Journal of the National Comprehensive Cancer Network..

[3] Cejas P, Drier Y, Dreijerink KM, Brosens LA, Deshpande V, Epstein CB (2019). Enhancer signatures stratify and predict outcomes of non-functional pancreatic neuroendocrine tumors. Nature medicine..

[4] The Chicago consensus on peritoneal surface malignancies: management of neuroendocrine tumors Annals of surgical oncology.

[5] Barthet M, Giovannini M, Lesavre N, Boustiere C, Napoleon B, Koch S (2019). Endoscopic ultrasound-guided radio-frequency ablation for pancreatic neuro-endocrine tumors and pancreatic cystic neoplasms: a prospective multicenter study. Endoscopy..

[6] Lv Y, Han X, Xu X-F, Ji Y, Zhou Y-H, Sun H-C (2019). Risk factors affecting prognosis in metachronous liver metastases from WHO classification G1 and G2 gastroentero-pancreatic neuroendocrine tumors after initial R0 surgical resection. BMC cancer..

[7] Mazzaferro V, Pulvirenti A, Coppa J (2007). Neuro-endocrine tumors metastatic to the liver: how to select patients for liver trans-plantation?. Journal of hepatology..

[8] De Jong M, Breeman WA, Kwekkeboom DJ, Valkema R, Krenning EP (2009). Tumor imaging and therapy using radiolabeled somato-statin analogues. Accounts of chemical research..

[9] Krenning E, Kwekkeboom DJ, Bakker Wea, Breeman W, Kooij P, Oei H (1993). Somatostatin receptor scintigraphy with [111 In-DTPA-D-Phe 1]-and [123 I-Tyr 3]-octreotide: the Rotterdam experience with more than 1000 patients. European journal of nuclear medicine..

[10] Simons ZB, Wangsiricharoen S, Gelwan E, Lilja SB, Santhanam P (2020). SUN-904 Ga68 Dotatate Detects Ectopic ACTH Secreting Atypical Carcinoid Tumor. Journal of the Endocrine Society..

[11] Hofman MS, Lau WE, Hicks RJ (2015). Somatostatin receptor imaging with 68Ga DOTATATE PET/CT: clinical utility, normal patterns, pearls, and pitfalls in interpretation. Radiographics..

[12] Guenter R, Aweda T, Matos DMC, Jang S, Whitt J, Cheng Y-Q (2020). Overexpression of somatostatin receptor type 2 in neuro-endocrine tumors for improved Ga68-DOTATATE imaging and treatment. Surgery..

[13] Ćwikła JB, Bodei L, Kolasinska-Ćwikła A, Sankowski A, Modlin IM, Kidd M (2015). Circulating transcript analysis (NETest) in GEP-NETs treated with somatostatin analogs defines therapy. The Journal of Clinical Endocrinology & Metabolism..

[14] Melis M, Krenning EP, Bernard BF, Barone R, Visser TJ, de Jong M (2005). Localisation and mechanism of renal retention of radio-labelled somatostatin analogues. European journal of nuclear medicine and molecular imaging..

[15] Li WP, Lewis JS, Kim J, Bugaj JE, Johnson MA, Erion JL (2002). DOTA− d-Tyr1-Octreotate: A Somatostatin Analogue for Labeling with Metal and Halogen Radionuclides for Cancer Imaging and Therapy. Bioconjugate chemistry..

[16] Van Essen M, Krenning EP, De Jong M, Valkema R, Kwekkeboom DJ (2007). Peptide receptor radionuclide therapy with radiolabelled somatostatin analogues in patients with somatostatin receptor positive tumours. Acta Oncologica..

[17] Bombardieri E, Maccauro M, de Deckere E, Savelli G, Chiti A (2001). Nuclear medicine imaging of neuroendocrine tumours. Annals of Oncology..

[18] Bozkurt MF, Virgolini I, Balogova S, Beheshti M, Rubello D, Decristoforo C (2017). Guideline for PET/CT imaging of neuroendocrine neoplasms with (68)Ga-DOTA-conjugated somatostatin receptor targeting peptides and (18)F-DOPA. Eur J Nucl Med Mol Imaging..

[19] Gustafsson BI, Kidd M, Modlin IM (2008). Neuro-endocrine tumors of the diffuse neuro-endocrine system. Current opinion in oncology..

[20] Modlin IM, Oberg K, Chung DC, Jensen RT, de Herder WW, Thakker RV (2008). Gastro-enteropancreatic neuroendocrine tumours. The lancet oncology..

[21] Crown A, Rocha FG, Raghu P, Lin B, Funk G, Alseidi A (2020). Impact of initial imaging with gallium‐68 dotatate PET/CT on diagnosis and management of patients with neuroendocrine tumors. Journal of Surgical Oncology..

[22] Begum N, Maasberg S, Plöckinger U, Anlauf M, Rinke A, Pöpperl G (2012). Neuroendocrine tumours of the GI tract--data from the German NET Registry. Zentralblatt fur Chirurgie..

[23] Hofman MS, Kong G, Neels OC, Eu P, Hong E, Hicks RJ (2012). High management impact of Ga-68 DOTATATE (GaTate) PET/CT for imaging neuroendocrine and other somatostatin expressing tumours. J Med Imaging Radiat Oncol..

[24] Özkan ZG, Kuyumcu S, Balköse D, Ozkan B, Aksakal N, Yılmaz E (2013). The value of somatostatin receptor imaging with In-111 Octreotide and/or Ga-68 DOTATATE in localizing Ectopic ACTH producing tumors. Mol Imaging Radionucl Ther..

[25] Frilling A, Sotiropoulos GC, Radtke A, Malago M, Bockisch A, Kuehl H (2010). The impact of 68Ga-DOTATOC positron emission tomography/ computed tomography on the multimodal management of patients with neuro-endocrine tumors. Annals of surgery..

[26] Yang J, Kan Y, Ge BH, Yuan L, Li C, Zhao W (2014). Diagnostic role of Gallium-68 DOTATOC and Gallium-68 DOTATATE PET in patients with neuroendocrine tumors: a meta-analysis. Acta Radiol..

[27] Delpassand ES, Ranganathan D, Wagh N, Shafie A, Gaber A, Abbasi A (2020). 64Cu-DOTATATE PET/CT for Imaging Patients with Known or Suspected Somatostatin Receptor–Positive Neuroendocrine Tumors: Results of the First US Prospective, Reader-Masked Clinical Trial. Journal of Nuclear Medicine..

